# Comparison of the effectiveness of thoracic epidural steroid and local anesthetic injections: A randomized controlled trial

**DOI:** 10.1097/MD.0000000000045994

**Published:** 2025-11-14

**Authors:** Dostali Aliyev, Derya Bayram, İbrahim Aşik

**Affiliations:** a Department of Anesthesiology and Reanimation, Division of Algology, Tobb Etü Hospital, Faculty of Medicine, Ankara, Turkey; b Ministry of Health Mardin Training and Research Hospital, Pain Clinic, Mardin, Turkey; c Department of Anesthesiology and Reanimation, Division of Algology, Ankara University, Faculty of Medicine, Ankara, Turkey.

**Keywords:** chest wall pain, chronic thoracic pain, steroid, thoracic interlaminar epidural injections

## Abstract

This study primarily aims to evaluate the effectiveness of thoracic interlaminar epidural injections in alleviating pain and enhancing function in patients with chronic mid and/or upper back pain. One hundred patients were randomly divided into 2 groups, 50 patients in each. Group I received only a local anesthetic, whereas Group II received a combination of local anesthetic and steroids. The random assignment to either Group I or Group II was determined using a simple computer-generated sequence. The results were evaluated using the numeric rating scale and the revised Oswestry Disability Index. Patients who showed significant improvement for >4 weeks after the first 2 procedures were considered successful. Those who did not were classified as failed participants. Significant improvement was defined as a reduction by >50% in both numeric rating scale and revised Oswestry Disability Index scores, and assessments were conducted at the beginning and 3rd, 6th, and 12th months after the intervention. This study included 100 participants divided into 2 groups. At the 12th month, significant pain relief (≥50%) and a reduction of at least 50% in ODI scores from baseline were observed in 86% of patients in Group I and 90% of patients in Group II. The results achieved in this study suggest that chronic thoracic pain of non-facet joint origin can be managed conservatively using thoracic interlaminar epidural injections, either with or without steroids.

## 1. Introduction

Thoracic pain affects approximately 13% of individuals, but chronic mid and upper back pain is relatively rare in comparison to low back and neck pain.^[1,2]^ Despite its low frequency, thoracic spine pain can lead to as much disability as pain from other spinal regions.^[3]^

The application of epidural injections in treating chronic thoracic pain, particularly the ones caused by disc herniation, radiculitis, axial or discogenic pain, spinal stenosis, and other thoracic spine abnormalities, as well as postoperative and post-thoracotomy pain syndromes, is not well-documented in medical literature. Even though the exact mechanisms through which epidurally administered steroids and local anesthetics provide relief are not fully understood, they are thought to offer sustained pain relief through various pathways.^[4]^ Specifically, local anesthetics may affect several pathophysiological processes involved in chronic pain, such as peripheral noxious stimulation and the sensitization of pain pathways, along with the overproduction of neurotransmitters.^[5]^

The present study aims to investigate the effect of fluoroscopy-guided epidural drug (steroid, steroid + local anesthetic) injection on chronic back pain.

## 2. Materials and methods

This study was carried out in a single center in Ankara-Türkiye, between the dates of August 15, 2021 and November 01, 2022 after the approval of the ethics committee (date: August 12, 2021 and protocol number: I7-487-21). The Declaration of Helsinki was followed throughout the study process, and the confidentiality of patient data was guaranteed. The study was registered with the U.S. Clinical Trial Registry with an assigned number of NCT06507995.

The research included patients visiting the pain clinic for upper and mid-back pain, and all participants provided written informed consent after receiving detailed information about the study. Exclusion criteria included individuals under 18, those with psychological disorders, allergies to local anesthetics or steroids, thoracic facet joint pain, severe disc herniation, or signs of infection, which were ruled out by pre-procedure blood tests and C-reactive protein levels.

Inclusion criteria include being older than 18 years, having suffered from chronic mid and upper back pain for >6 months, and having not responded to conservative management, including medication, physical therapy, and exercise programs, and having no facet joint pain confirmed through diagnostic blocks.

The participants (N = 100) were evenly divided into 2 groups. Group I received thoracic interlaminar epidural injections of 6 mL of 2% preservative-free prilocaine. Group II received injections of 4 mL of 2% preservative-free prilocaine combined with 2 mL or 8 mg of dexamethasone, totaling 6 mL. Injections were administered under sedation and sterile conditions, and all patients were positioned prone and monitored. A single physician performed all injections using an 18-gauge Tuohy needle, employing the loss of resistance technique and confirming the epidural space placement with 5 mL of nonionic contrast. The injection sites were chosen considering the pain reported by the patient and corroborated by clinical and radiological findings.

Follow-up injections were considered based on initial response, and additional interventions were provided for those reporting <50% pain relief and functional decline. All participants maintained their usual medication regimen and physical activity, including work. The present study aims to assess the efficacy of thoracic interlaminar epidural injections, with or without steroids, by using fluoroscopy to manage chronic pain in the mid and upper back, excluding facet joint pain. The results were measured at the beginning and at 3rd, 6th, and 12th months by using pain scores and functional status via NRS (numeric rating scale) and rODI (revised Oswestry Disability Index) scales.^[6]^

Patients were randomly assigned to either Group I or II using a simple computer-generated sequence. The drugs were prepared by an individual not involved in administering interventions, and the group assignment was blinded to all, including the administering physician and patients. Both injection solutions had a clear appearance, which makes group assignments indiscernible. The participating patients were also integrated with others receiving routine spinal interventions, and the nature of the participants was not disclosed to the performing physician.

## 3. Results

The demographic characteristics of the study participants are presented in Table 1. There were no significant differences between the groups in terms of age and gender. While there were variations in height and weight, these differences were not statistically significant when evaluated based on body mass index.

**Table 1:** Baseline demographic characteristics.

	Group Ⅰ Mean ± SD	Group Ⅱ Mean ± SD	*P*
Gender			.265
Women	78% (n = 39)	66% (n = 33)	
Men	22% (n = 11)	34% (n = 17)	
Age	64.68 ± 12.76 (min 38–max 86)	62.54 ± 15.60 (min 25–max 92)	.455
Weight	63.88 ± 11.64 (min 47–max 99)	68.98 ± 12.08 (min 48–max 100)	.034
Height	158.98 ± 7.64 (min 150–max 175)	162.48 ± 6.57 (min 150–max 182)	.016
Body mass index	30.41 ± 34.85 (min 19.3–max 27)	26.19 ± 4.40 (min 19.0–max 36.7)	.399
Duration of pain (months)	94.32 ± 20.10 (min 63–max 150)	96.08 ± 17.43 (min 65–max 135)	.641
Diagnosis			.544
Discogenic*	46% (n = 23)	38% (n = 19)	
Disc herniation	54% (n = 27)	62% (n = 31)	

Mean ± SD, median, independent *t* test, paired *t* test, *P* < .05.

* Discogenic pain, stenosis, etc.

The study included 100 participants, with Group 1 receiving only local anesthetic injections, and Group 2 receiving both local anesthetic and steroid injections. Pain levels were monitored over a 1-year period, and assessments were made at the beginning of the intervention and then at 3rd, 6th, and 12th months after the intervention. The results revealed statistically significant reductions from baseline for both the NRS and rODI in both groups, with *P*-values of .000 for both scales. Comparing the groups, the results indicated that pain decreased significantly in both Group 1 and Group 2, but the results were in favor of patients who received combination therapy (*P* = .000). However, there was no significant difference between the rODI scores of groups (*P* = .790). The results indicate a significant improvement when compared to baseline and were similar, as shown in Table 2.

**Table 2:** Comparison of the numeric pain rating scale and the revised Oswestry Disability Index (rODI) scores for 1 year.

Time points	NRS		rODI	
	Group Ⅰ	Group Ⅱ	Group Ⅰ	Group Ⅱ
	Mean ± SD	Mean ± SD	Mean ± SD	Mean ± SD
Baseline	8.76 ± 0.91	8.46 ± 0.99	45.54 ± 5.17	47.30 ± 4.50
3 months	4.56 ± 0.57	4.58 ± 0.57	22.66 ± 2.73	23.24 ± 2.70
6 months	3.66 ± 0.55	3.42 ± 0.75	22.48 ± 2.43	22.66 ± 2.17
12 months	3.46 ± 0.78	2.78 ± 0.76	22.86 ± 2.29	22.98 ± 2.19
Time difference	.000		.000	
Group difference	.000		.790	

Mean ± SD, median, repeated measures, chi-square test, *P* < .05.

The criteria for a successful patient outcome included a significant improvement at least 4 weeks after the first 2 injections. On the other hand, the patients who did not experience pain relief lasting at least 4 weeks after the first 2 injections were considered to indicate an unsuccessful intervention. Detailed epidural injection characteristics for both groups, including the numbers of injections and weeks of pain relief, are shown in Table 3.

**Table 3:** Epidural procedural characteristics with average relief in weeks over a period of 1 year for thoracic pain.

	Successful patients		Failed patients		All patients		
	Group 1 (43)	Group 2 (45)	Group 1 (7)	Group 2 (5)	Group 1 (50)		Group 2 (50)
*At 1 yr*							
Average number of procedures per year	3.6 ± 1.1	3.5 ± 1.0	1.6 ± 0.6	2.4 ± 15		3.4 ± 1.2	3.5 ± 1.3
Total number of procedures per year	168	171	12	10		180	181
Total relief per year (wk)	42.2 ± 10.4	44.6 ± 11.0	1.6 ± 1.7	2.1 ± 2.5		37.2 ± 16.5	41.6 ± 16.4

Successful patients: at least 4 weeks of significant improvement with the first 2 injections.

Of the 361 thoracic interlaminar epidural procedures performed, there was 1 subarachnoid puncture. No postoperative headache was reported. Three patients developed immediate postoperative pain and spasm, lasting for 3 hours.

### 3.1. Statistical analysis

Data analysis was conducted utilizing SPSS version 24.0 (SPSS Inc., Chicago). Chi-square tests (or Fisher exact test when needed) and *t* tests were applied to compare categorical and continuous variables, respectively. Fisher exact test was utilized for instances where the expected count was below 5. The *t* test was employed for comparing mean scores between groups, while paired *t* tests were used to evaluate changes in average pain scores and rODI from baseline and at the follow-up periods of 3, 6, and 12 months. Statistical significance was set at *P* < .05. Sample size was calculated using G-Power 3.1 (Heinrich-Heine-University Düsseldorf, North Rhine-Westphalia, Germany) at a power (1-β) level of 0.80 and a confidence level of 95%, which indicated a sample size of 31 participants per group.

## 4. Discussion

This study aims to evaluate the effectiveness of thoracic interlaminar epidural injections for treating chronic mid- and upper-back pain due to conditions such as disc herniation, spinal stenosis, radiculitis, or discogenic pain. Twelve months after the intervention, both groups reported statistically significant reductions in their Visual Analog Scale (VAS) and rODI scores. Notably, patients receiving the combined therapy of local anesthetic and steroids had better results in VAS scores.

The results demonstrated comparable efficacy between the use of the local anesthetic solely and in combination with steroids in terms of improving disability scores by the end of the 12 months. Specifically, 86% of patients who received only local anesthetic and 90% of those treated with both steroid and local anesthetic reported significant improvements in their condition, as detailed in Table 3.

Even though it is less common, thoracic pain can severely hinder daily activities due to its debilitating nature. Thoracic epidural steroid injections have been widely performed to manage such pain, particularly for those related with herniated discs or spinal stenosis.^[7]^ Supported by evidence from randomized controlled trials, these injections are recognized as a safe and minimally invasive approach aiming to alleviate thoracic pain and enhance quality of life.

Three primary methods are employed to access the thoracic epidural space: caudal, interlaminar, and transforaminal epidural injections.^[4,8]^

The interlaminar approach, known for being less technically demanding, typically results in fewer side effects in comparison to the transforaminal approach. However, the latter may provide a more extensive spread of the injectate to the anterior epidural space, where pro-inflammatory substances are often present.^[9]^

Unlike the cervical and lumbar spine, performing an interlaminar epidural injection in the thoracic region poses additional challenges due to the proximity of the lungs, ribs, and the angled orientation of the spinous processes. A midline approach may be impractical in case of the pain localized to one side; in these instances, an ipsilateral paramedian approach can be more effective.

The paramedian interlaminar approach was used in this study. By opting for a parasagittal interlaminar route instead of a direct midline path, there is potential for enhanced drug delivery to the anterior epidural area. This technique involves directing the needle laterally within the interlaminar space, closer to the nerve roots, which can lead to better pain management outcomes. Previous studies indicated that the parasagittal interlaminar approach can improve both pain and functional disability scores in comparison to the traditional midline method.^[10]^

The results achieved in this study also reflected a favorable impact of the paramedian interlaminar approach, whether using local anesthetics with or without steroids, on both VAS and disability scores.

Effective management of thoracic pain necessitates careful evaluation of various factors, including the precise location for accessing the epidural space and employing the correct techniques. It is very important to accurately determine the specific origin of pain and the most appropriate intervention site for successful outcomes. The access point to the epidural space should be selected by considering the patient’s specific pain complaints, as well as clinical and radiological evidence. Furthermore, to maximize efficiency, it is recommended to perform the intervention at or below the level indicated by the patient’s symptoms.

Even though epidural steroid injections are generally considered safe, they must be administered for appropriate indications due to the potential severity of complications. As such, it is strongly recommended to perform these injections under fluoroscopic guidance. This approach helps reduce the risk of complications and enhances the safety and effectiveness of the intervention.^[9]^

The precise mechanisms behind the effects of epidural anesthetics and steroids remain partially understood.^[11,12]^

It is hypothesized that both local anesthetics and steroids offer durable relief through various pathways, including the suppression of ectopic nerve discharges, improvement of blood flow to ischemic nerve roots, breakdown of iatrogenic and inflammatory adhesions, removal of pro-inflammatory cytokines, and the reversal of both peripheral and central sensitization.^[13–16]^

Numerous studies comprehensively assessed the efficacy of epidural injections using saline, local anesthetics, and steroids.^[17,18]^

The most frequent combinations employed are local anesthetics either alone or combined with steroids, steroids alone, or steroids mixed with saline. Yet, the ideal combination and dosage have not been conclusively established yet.^[19]^

Studies on the use of steroids and local anesthetics, either alone or in combination, in epidural injections reported mixed results. Some studies reported no difference in outcomes, while others indicated significant differences. Evidence suggested that injections containing local anesthetics with or without steroids could remarkably reduce both acute and chronic pain.^[20]^ Specifically, steroids were reported to offer quicker, more effective, and longer-lasting pain relief in comparison to local anesthetics alone for conditions like disc herniation. Some findings also suggested that the efficacy of local anesthetics alone is comparable to their use with steroids across various spinal conditions.^[8]^ Moreover, certain studies emphasized that adding corticosteroids to local anesthetics could enhance pain relief across all types of approaches. Systematic reviews and meta-analyses revealed that lidocaine, either alone or in combination with steroids, effectively treats lumbar disc herniation and spinal stenosis.^[21,22]^

In this research, both the use of a combination of steroids and local anesthetics and the exclusive use of local anesthetics led to significant reductions in VAS and disability scores after a 12-month follow-up. Even though no significant differences were found in disability scores between the 2 groups, patients receiving the combined intervention had better outcomes in VAS scores.

A study involving 40 patients assessed the effectiveness of thoracic interlaminar epidural injections, revealing that 80% of the participants in Group I, who received only local anesthetic, and 85% of those in Group II, who were treated with both local anesthetics and steroids, reported significant improvements in functional status and pain relief of at least 50%. Over the course of a year, no significant differences were observed between the 2 groups. It was concluded that, provided thoracic facet joint arthropathy is excluded as a cause of chronic pain, employing local anesthetics alone or in combination with steroids presents a viable option for managing thoracic pain.^[5]^

Corticosteroids used in epidural injections include triamcinolone acetonide, betamethasone sodium phosphate/betamethasone acetate, and dexamethasone sodium phosphate. It is very important to consider that particulate steroids pose a higher risk of complications than non-particulate ones. The accidental intravascular injection of particulate steroids can increase the likelihood of ischemic events. Instances of spinal cord ischemia and posterior circulation infarction following cervical epidural injections were reported.^[20]^ Therefore, it is generally not recommended to use particulate steroids in epidural injections above the L2 vertebra.

Non-particulate steroids, such as dexamethasone, are considered a safer choice for epidural injections. Dexamethasone, a non-particulate steroid recommended for epidural use, was utilized in this study. While a study carried out in 2016 reported particulate steroids to be more effective in reducing pain when compared to non-particulate steroids, the U.S. Food and Drug Administration in 2011 advised against using triamcinolone for epidural steroid injection.^[23,24]^ Moreover, a meta-analysis indicated that particulate steroids do not offer superior pain relief over non-particulate steroids, recommending the use of non-particulate options.^[25]^

Mid and upper back pain can be severely debilitating for many individuals. To enhance the effectiveness of the intervention, some patients may require repeated procedures. A recent study evaluated the effectiveness of multiple thoracic interlaminar epidural steroid injections (an average of 5 to 6 injections) over a 2-year period in patients suffering from mid and upper back pain caused by disc herniation, radiculitis, or discogenic pain. The study involved 110 patients divided into 2 groups: one group received only local anesthetic, and the other group received a combination of local anesthetic and steroids. Even though there was no placebo group, their findings were significant. The study reported that 71% of patients receiving only local anesthetic and 80% of those receiving the combination therapy showed improvement. It suggested that thoracic interlaminar epidural steroid injections could be an effective intervention for many individuals. Patients were categorized into nonresponsive and responsive groups, with a successful outcome defined as significant improvement (>50%) observed 3 weeks after the first 2 procedures. At the 2-year follow-up, 80% and 86% of patients in each intervention group, respectively, experienced successful outcomes, reporting improved pain and functional status along with a significant reduction in opioid usage. The complication rate was low, with only 4 cases reported: 2 subarachnoid punctures, one instance of post-procedure pain at the injection site, and one case of lower extremity pain.^[7]^

In the present study, repeat thoracic epidural steroid injections were administered to some patients, and it was noted that those who showed only a partial response initially experienced >50% pain reduction after the second procedure.

Both transforaminal and interlaminar epidural steroid injections are considered safe when performed in accordance with evidence-based guidelines. However, the potential for adverse outcomes from thoracic epidural injections was investigated in relatively few studies. Reported complications and side effects include pain at the injection site, facial flushing, headache, insomnia, fever on the night of the procedure, accidental intravascular entry, significant bleeding, local hematoma, bruising, vasovagal reactions, transient nerve root irritation, and transient spinal cord irritation.^[26–28]^

It is important to acknowledge that the risk of complications may differ by the injection technique used. For instance, vasovagal reactions tend to occur more frequently with transforaminal injections, whereas dural punctures are more common with interlaminar approaches. Despite these immediate concerns, no significant differences in rates of delayed minor adverse events were noted, and no long-term sequelae were observed in any of the minor adverse events among the patients participating in the present study. When administered correctly, epidural steroid injections can be an effective method for managing pain and alleviating discomfort.

Epidural injections that utilize local anesthetics carry higher risks, particularly when administered in the upper thoracic and cervical regions. Severe complications were reported, including paralysis and death, resulting from cervical and thoracic transforaminal epidural injections where only local anesthetics were used.^[29–31]^

Additionally, patients with a history of previous surgeries are considered to be at an even higher risk of experiencing these severe complications.^[8]^

Epidural injections can be useful in reducing opioid addiction and the need for surgery in patients experiencing severe pain and dysfunction. However, thoracic epidural injections are not the sole intervention option for thoracic pain. If thoracic epidural steroid injections do not sufficiently alleviate the pain, surgical interventions may be considered as an alternative for some patients.

This study has several limitations, including a small sample size that could affect the generalizability of the results. Assessing thoracic pain can be challenging due to the often vague and undefined nature of the symptoms. Despite these issues, the present study included only patients who met specific inclusion criteria, and data were collected prospectively. Additionally, this study did not include a sham-treated control group, which could impact the robustness of the conclusions. On a positive note, no serious injection-related complications or side effects were reported among the participating patients. Furthermore, the present study evaluated the efficacy of the intervention across short-, mid-, and long-term periods following the procedure.

In conclusion, this study aims to assess the effectiveness of thoracic interlaminar epidural injections, either with local anesthetic alone or combined with steroids, in alleviating chronic mid and upper back pain due to various conditions such as disc herniation, radiculitis, axial or discogenic pain, and spinal stenosis. The results clearly showed that both groups of patients experienced significant improvement, as evidenced by NRS and revised rODI scores, during 12 months of follow-up after the intervention. Notably, those receiving combination therapy had particularly better outcomes in NRS scores. Both intervention modalities had a similar impact on disability in the short and long term. A significant proportion of patients in each group reported remarkable improvements; 86% of those who received only local anesthetic and 90% of those treated with both steroids and local anesthetic experienced substantial relief.

## 5. Conclusion

These results suggested that thoracic epidural injections could be an effective alternative for patients suffering from chronic mid and upper back pain and disability. The interventions not only carried a lower risk of complications and side effects but also offered significant relief for a range of conditions, including disc herniation, radiculitis, and spinal stenosis. Despite these encouraging outcomes, there still is a need to standardize the approach to interlaminar epidural injections, particularly concerning the type and dosage of injectate, timing for reinjections, and clear indications for use. Future research with larger sample sizes is essential to provide definitive recommendations and draw more robust conclusions.

## Author contributions

**Conceptualization:** Dostali Aliyev, Derya Bayram, İbrahim Aşik.

**Data curation:** Dostali Aliyev.

**Formal analysis:** Dostali Aliyev, Derya Bayram, İbrahim Aşik.

**Investigation:** Dostali Aliyev.

**Methodology:** Dostali Aliyev.

**Project administration:** Dostali Aliyev.

**Resources:** Dostali Aliyev.

**Software:** Dostali Aliyev.

**Supervision:** Dostali Aliyev.

**Validation:** Dostali Aliyev.

**Visualization:** Dostali Aliyev.

**Writing – original draft:** Dostali Aliyev, Derya Bayram, İbrahim Aşik.

**Writing – review & editing:** Dostali Aliyev, Derya Bayram, İbrahim Aşik.
